# Recommending Physical Activity During the COVID-19 Health Crisis. Fitness Influencers on Instagram

**DOI:** 10.3389/fspor.2020.589813

**Published:** 2020-12-03

**Authors:** Joseph Godefroy

**Affiliations:** Department of Sociology, Laboratory CENS CNRS 6025 (Centre Nantais de Sociologie), Université de Nantes, Nantes, France

**Keywords:** instagram, influencers, fitness, COVID-19, social media, platform capitalism, sociology

## Abstract

Fitness content creators on Instagram used the COVID-19 lockdown period to strongly and frequently recommend physical activity to their followers. These individuals are not professional fitness trainers and their Instagram activity consisted of sharing images that were more about the staging of their bodies than about educational content. However, when fitness clubs in France were forced to close in March 2020 following the government's decision to restrict non-essential movement and activities, influencers changed the images they shared daily to promote fitness training that could be done at home. In comparison, this study also analyses the case of a chain of fitness clubs which offered live fitness sessions online via its Instagram account, in order to manage the repercussions of the forced closure of its establishments. This article reveals some consequences of the temporary lockdown of the fitness training industry in France and questions the new dynamic of online fitness recommendations that was observed during the COVID-19 pandemic.

## Introduction

The COVID-19 pandemic has resulted in an unprecedented global upheaval as regards physical activity (Table 1). Major sporting events, such as the Olympic Games have been postponed, access to many public sports facilities has been closed and sports businesses, unable to function normally, face bankruptcy in many cases. This period has also seen the development of physical training at home and online. Through the study of the activity of fitness influencers during this period, and of a chain of fitness clubs that offered live fitness sessions online via its Instagram account, this article reports on the consequences of the shutdown of the fitness training industry in France and questions the new dynamic of online fitness recommendations that was observed during the COVID-19 pandemic.

**Table 1:** The French national regulations on physical activities during the COVID-19 pandemic, between 14 February and 2 June, 2020.

**The COVID-19 health crisis in France**	
01/09/2020	First official COVID-19-related death in Wuhan, China.
02/14/2020	First death recorded on French soil.
02/25/2020	First French person to die from COVID-19.
03/11/2020	COVID-19 declared to be a pandemic by the WHO.
03/14/2020	French Prime Minister Edouard Philippeannounces the closure of all establishments open to the public and deemed “not essential” to the life of the country. Closing of all fitness clubs.
03/16/2020	French President Emmanuel Macron announces travel restrictions in the country. Individual physical activity is allowed but must be “brief.” Moreover, in consultation with other European leaders, the borders of the Schengen area are closed.
03/23/2020	Prime Minister Edouard Philippe announces that individual physical activity is to be limited to 1 h per day within a radius of 1 km (0.6 mile) of the home.
04/07/2020	The Prefecture and City Hall of Paris bans individual fitness activities between 10 a.m. and 7 p.m. in all outdoor public spaces.
05/11/2020	Lockdown is lifted but some restrictions remain in place. Fitness clubs are still closed.
06/02/2020	Reopening of establishments open to the public. Reopening of fitness clubs.

Recent French sociological research has focused on digital spaces that allow consumers and workers to interact (Beauvisage et al., 2018; Chaves Ferreira, 2018; Jan, 2018; Jourdain, 2018). Different from these media, the social network Instagram, a non-market platform^1^, saw the development of a new way of working (Woodcock and Graham, 2018). The present research uses an ethnographic approach in order to question the boundaries of work in the era of digital platform capitalism (Cardon and Casillia, 2015; Srnicek, 2017; Abdelnour and Bernard, 2018; Abdelnour and Meda, 2019; Woodcock and Graham, 2020), with a particular focus on creators specialized in fitness content (or fitness influencers as they are commonly known). It investigates a French fitness center and fitness influencers, keen to find alternative ways of promoting physical activities during the COVID-19 lockdown, such as offering live online fitness instruction. This article identified the different interactions established between how individuals and organizational fitness influencers used Instagram before and during the pandemic.

By identifying their ways of working through and with Instagram, this research shows that influencers' activity reflects the interplay of production and consumption in their everyday use of online social networks (Ritzer and Jurgenson, 2010). In the same way as the highly commodified and spectacularized world of professional sport, by engaging fans via their Instagram accounts, influencers can open new communication channels with their audience that can be measured and valued as a new commercial opportunity for sponsors (Cave and Miller, 2015).

Moreover, the activity analyzed here cannot be fully understood without recourse to the literature of the sociology of the body. This study reveals that health and fitness issues are at the heart of the practices exhibited on Instagram by fitness influencers (Smith Maguire, 2008). By inciting their followers to work out and by giving them precise instructions within a fun framework, the individuals observed adopted the role of “sports coach,” disseminating health and fitness instruction but without monitoring how they were understood or the way in which their instructions were carried out. In this sense, the qualitative analysis of the content shared by the respondents during the lockdown provides an additional key to understanding the dissemination of health and fitness recommendations during the COVID-19 pandemic.

The lockdown instigated in response to the COVID-19 crisis forced the fitness influencers studied, who were more used to the staging of their bodies than to the transmission of educational content, to change the images they shared daily on digital social media to include fitness sessions that could be done at home. In the same way, to manage the repercussions of their forced closure, fitness clubs started using social media to broadcast “live” fitness sessions. Far from resembling usual forms of fitness training, the productions by these two types of actors helped to build a new digital space that multiplied incentives to work out.

The primary aim of this study is to present empirical evidence of ways Instagram has been used as a tool to disseminate and encourage sports participation during the COVID-19 pandemic in France. During this period, online physical training content has been more available than ever. Our study shows that the role played by social medial and its users during the quarantine can be understood through the notion of “prosumer”.

## Method

This article draws on sociological research conducted since 2017 which studies the economic and professional uses of digital social media. An ethnographic approach (Miller, 2011; Luvaas, 2012; Hjorth et al., 2017) to the research was adopted and included fifty semi-structured interviews^2^ conducted with influencers on Instagram. An influencer could be selected for the research if he or she was an Instagram user publishing content exclusively dedicated to fitness with at least one commercial partnership with a company. Furthermore, a qualitative analysis was performed of the content shared on Instagram by the influencers encountered, of observations at events featuring influencers and the companies sponsoring them, and of participant observations (Wacquant, 2004) during training sessions organized and led by Facebook and Instagram. Finally, this study also included a quantitative analysis of income received by influencers through certain partnerships. This research context has allowed us to understand the way in which influencers organize and carry out their activity. The approach adopted here focuses more precisely on the consequences related to the shutdown of the fitness training industry in France.

The analysis presented in this paper is based on a corpus of elements (images, videos, texts, comments) collected during the first 6 weeks of the lockdown. For this purpose, after inspecting each of the images and videos published by the respondents during this period, the online textual data^3^ was analyzed. Moreover, the number of the influencers' and gym's Instagram followers was extracted on the first and last day of lockdown to see if the COVID-19 pandemic had affected their audience.

## Results

### The Production of New Content on Instagram

Faced with the closure of fitness clubs, which had been until then the setting favored by fitness influencers to build the content they shared online, the individuals observed seem to have given an additional dimension to the health and fitness recommendations they previously shared, which were often no more than the (generally unconscious) advocating of a specific ideal body type on the Internet. The observation of this digital space thus allowed us to understand a new practice developing among individuals who, until that point, had limited the nature of their posts to the staging of their bodies (Détrez, 2002). During lockdown, the fitness influencers observed started teaching physical exercises that could be done at home. Although they were not fitness training professionals, these women and men used the lockdown period to offer a “*special lockdown workout routine*” often containing exercises that did not require “*fitness equipment*.”

“Just because we're stuck at home doesn't mean we have to put our fitness on hold! Let's work out at home, for our physical and mental health, and to prepare that summer body.”^4^

(Excerpt from the Instagram post of Estelle,^5^ a French fitness influencer, 2 April 2020)

Fitness recommendations tended to take several forms. First of all, the previously “fixed” and meticulously staged content gave way to more dynamic video content showing the star-Instagrammer working out at home. The training instructions included in the video were only very rarely spoken aloud by the influencer. These short videos (Instagram limits the length of the videos published to 1 min) were, however, often accompanied by written and easily identifiable instructions. Alongside these iconographic supports (videos and photos), the “caption” function provided a space for free expression where an explanation of the instructions could be given so the exercises recommended could be performed correctly. These instructions were clear and gave the name of the exercises, the number of repetitions, the recovery time, and so on.

The lockdown period was also an opportunity for these fitness influencers to make the most of the previously little-used functionalities offered by the platform. Most of the individuals observed started sharing live content. These “lives” (daily, for some) then allowed a new mode of interaction with their followers and helped make physical exercise part of a certain routine. These online events never took the form of a reciprocal exchange between the content creator and his or her followers. Rather, the influencer took on the role of a coach and delivered live fitness sessions, without taking into account the reactions of their viewers. During these live posts, viewers could “comment” on what they were watching and send a message to the person sharing the video. The messages sent appeared in real time on a scrolling banner at the bottom of the video making it difficult for an influencer in the middle of demonstrating an exercise to take the scrolling messages into account. Semi-structured interviews conducted before the lockdown showed that the majority of respondents were not very comfortable with video staging. They preferred photos, because they felt more “*in control*” of the image they were showing. Although few respondents were willing to put video exercises online before lockdown, it should nevertheless be noted that those who did were almost exclusively men, despite the survey population being predominantly female. Despite being very rarely used prior to lockdown, the “live” function tended to become much more commonly mobilized by the population observed during this period.

“I hope that everything's okay for you. Like you, I am learning to deal with this harsh reality that is going to become our daily life for the next few weeks! So I'm pleased to officially announce a one-off upcoming ‘fit week’ comprising four sessions. A complete video and the schedule will be released tomorrow with information concerning the sessions. On the program: a live session on YouTube every other day for two weeks. And for the days in between, real-time videos posted online on Instagram. Sessions with and without equipment. Intensive, muscle-building, cardio, stretching and low impact sessions. So that's two weeks of non-stop training. An adapted and logically designed program to avoid doing any old thing and suffering unnecessary injuries. Let me remind you that these sessions will be sessions in their own right. Information will be given on each one. This is a huge challenge for me but also a great way to continue to have fun on my networks while saying thank-you to you. I look forward to working out with you, my team.”

(Excerpt from the Instagram post of Julie, a French fitness influencer, 18 March 2020).

“Just to remind you that this evening, like almost every day, there's going to be a big live event at 6:30 pm where I'll once again take immense pleasure in making you sweat and suffer! The advantage of this lockdown is that we're all going to be even fitter afterwards, right?”

(Excerpt from the Instagram post of Emma, a French fitness influencer, 1 April 2020)

By gradually incorporating this message into the daily lives of their Instagram followers, these individuals increasingly took on a role as an authority on the subject. By inciting their followers to work out with precise instructions within a fun framework, the individuals observed adopted the role of “sports coach,” disseminating health and fitness instructions but without checking how they were understood or the way in which they were carried out. Some differences were identified when comparing the instructional content of influencers and certified fitness coaches. For example, an influencer might present a fitness session with her dog in which she would execute an exercise using a large sack of dog food instead of weights. On the other hand, the certified fitness coach would insist on the right posture needed to perform the exercise without risk. The instructions of the influencers were less focused on the risks related to the technical correctness of the movements. Moreover, coaches from the fitness chain I studied insisted at the beginning of each training session on the type of public targeted by the nature of the training. For example, they specified that the “sculpt and hard cardio session” was not for “beginners,” “this training [was] for people who are already in good shape, experienced players.” While it is easy to report on the way this message was disseminated, it is difficult at this stage, for these individuals as well as for us, to grasp the real influence of these recommendations (Moreno Pestana, 2016).

“Being stuck at home can be an opportunity to work out and think about one's health. So here's my workout to do every day, to feel good and to maintain a regular fitness routine.”

(Excerpt from the Instagram post of Victoire, a French fitness influencer, 19 March 2020)

“You get to watch Netflix only after you've done 100 push-ups (in one or several goes depending on your level) and for those who are in good health and who can. If you want to come out stronger after this virus, you have to be able to do 100 push-ups non-stop. This will give you a minimum amount of daily exercise and a challenge to rise to. Take care of yourselves, and don't wait to magically get stronger someday – get stronger now!”

(Excerpt from the Instagram post of Steven, a French fitness influencer, 3 April 2020)

“Lockdown or not, this is the perfect opportunity to take the time to change your habits without the usual evenings out, without tempting dishes in restaurants, and regardless of your pathologies, disorders, diet or age. I will find the exercises that suit you. No excuses. On the contrary, now more than ever is the time to think about your immune system.”

(Excerpt from the Instagram post of Leslie, a French fitness influencer, 19 March 2020)

The interviews conducted prior to the COVID-19 health crisis underlined the need for informants to “stay active” on Instagram as much as possible. In this sense, almost all of the individuals interviewed published something at least once every other day.^6^ While they were deprived of access to fitness clubs, it seems reasonable to hypothesize that the practices described above were not only for educational purposes, but were also part of a strategy to remain visible (Mears, 2011). Indeed, these individuals need to be noticed in order to be contacted by brand names. To this end, influencers have to ensure that they produce regular content (Abidin, 2018). Forced to remain indoors, the staging of their physical fitness at home allowed them to maintain this rhythm.

By relying on wide visibility^7^ (Abidin, 2016), the individuals observed here convey a set of aesthetic criteria linked to the image of a muscular body which is staged in such a way as to be presented as an “ideal” (Smith Maguire, 2002). This dominant position on digital social media is usually seized by companies that see these individuals as a means of passing a subtle advertising message. Mobilized during the lockdown around the promotion of physical exercise at home, their bodies were put forward as the guarantees of the quality of the advice given, on a context where they were unable to fully monitor the effect of the exercises recommended. Away from professional training in the medical or sports field, it therefore seems that the legitimacy on which these individuals build their discourse is based on their previously constructed and staged physical appearance.

### Fitness Clubs Forced to Fall Back on Digital Technology

When the French Government prohibited the opening of fitness clubs on 14 March 2020, they were forced to suspend their activities. Confronted with the dissatisfaction of members who were demanding a refund of their membership fees, some brands found digital social media to be a way to create an offer within a certain continuity. Given the economic stakes related to the closure of their establishments (loss of earnings, loss of clients, etc.), some companies started offering a new service free of charge: the posting of live fitness training sessions on Instagram. Among these companies, the FitnessSide^8^ chain is a significant case that illustrates how Instagram has become a vector for health and fitness posts and demands during the pandemic.

Faced with the intensification of the number of cases and the progressive closure of certain companies, FitnessSide took a public stance by announcing on Friday 13 March the continued opening of its sports clubs and the adoption of specific measures to deal with the virus. The next day, when Prime Minister Edouard Philippe informed the country that all “*places open to the public that are not essential to the life of the country*” were to be closed, FitnessSide was forced, like all of its competitors, to announce the closure of its sports clubs. At the same time as this closure, the company redirected its clients to the existing My FitnessSideCoach application, presented at the time as “*your best ally to keep you motivated*.” In addition to online training sessions and advice, a message inviting clients to closely follow the brand's social networks was also sent out. Indeed, 3 days later, the company shared a publication on Instagram with the following text: “*Let's reinvent the way we move… at home!*”

“When the doors of the sports club, of the ‘temple’, of our happy ‘second home’, close. we need to totally reinvent our world… And that's exactly what we're going to do, together! Because human beings are endowed with a tremendous power of adaptation. If Sport is Life, then let's live! No question of giving up your good habits or resolutions.”

(Excerpt from the Instagram post on the FitnessSide account, 17 March 2020)

Following this announcement, and as in the case of the influencers observed, the FitnessSide Instagram account became the digital theater of fitness calls to action. The next day (18 March), the company announced its new training program to be followed live on Instagram: “*ConFITment*^9^ *by FitnessSide*.” This program was announced weekly and initially offered twice-daily online events allowing the live following of cardio training, yoga and muscle strengthening sessions (upper body, lower body or full body depending on the sessions).

“The daily grind is all in the past! We're moving on to the next level: integrating a little workout session whenever we can, either live with our great coaches or with the most useful application of the moment: My NeoCoach. The ConFITment is a good thing!”

(Excerpt from the Instagram post on the FitnessSide account, 19 March 2020)

The approach did not go unnoticed, forming part of a wider movement to which the televised evening news on *France 2* devoted a few minutes on Saturday 21 March. In a segment presenting “*the French people working out during lockdown*,” two FitnessSide coaches were shown giving a training session from their homes. This brief coverage also included an excerpt of an interview with a sports coach at FitnessSide, who explained how the company had “*tried to build content using the means at hand*” in order to allow people to work out with “*things that everyone usually has around their home*.”

**Figure 1:**
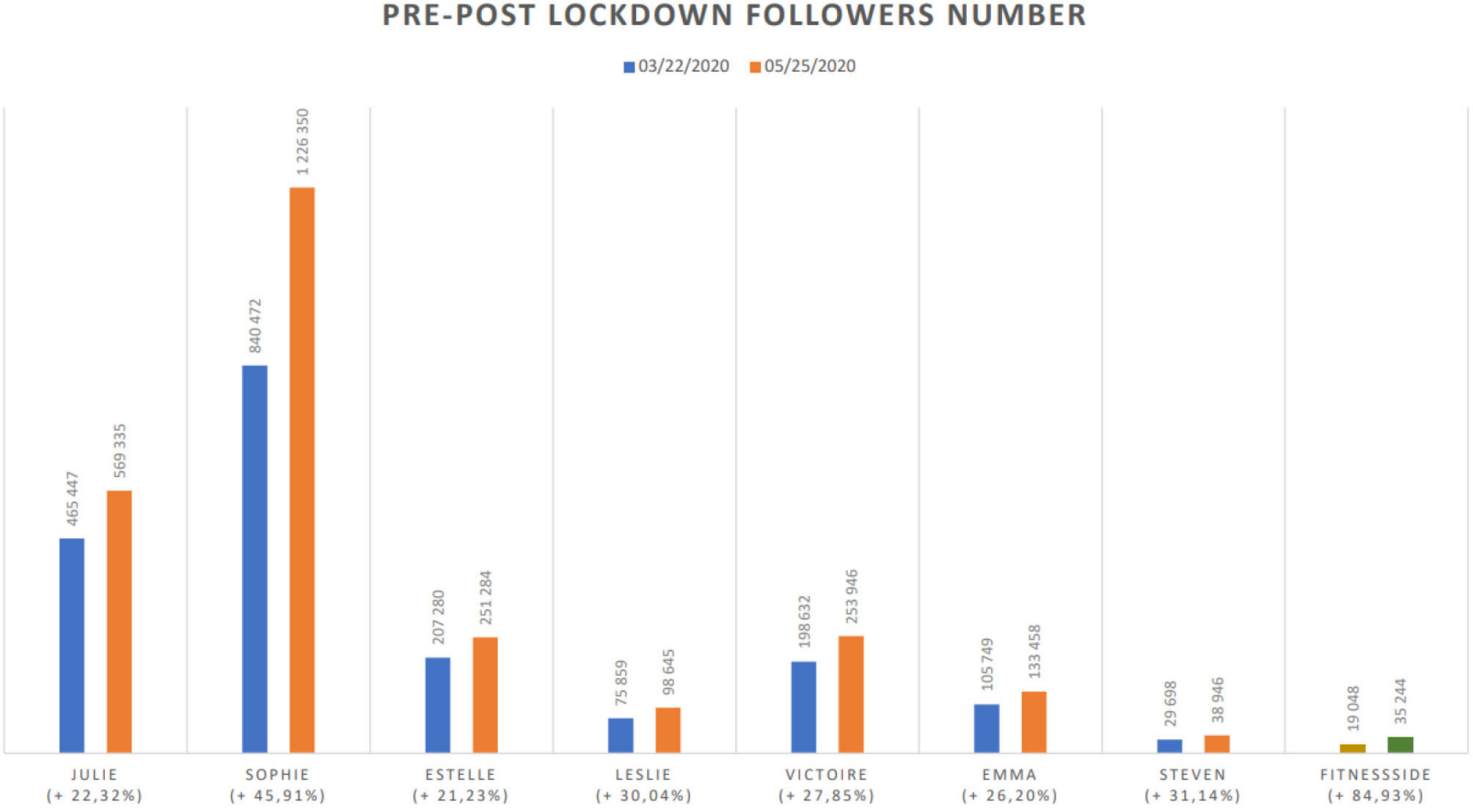
Pre-post lockdown followers number.

After 5 days of this program and 6 days of lockdown, FitnessSide reported that it had measured a high level of enthusiasm among its followers and was integrating new daily training slots. The second week of the program took the form of six sessions per day instead of two. In other words, the company increased its daily digital events by 200% to encourage fitness at home. In the same week as Prime Minister Edouard Philippe, at the instigation of the Council of State, limited the exemptions allowing physical activity to be maintained (23 March). On 10 March 2020 (6 days before the lockdown was announced), the FitnessSide Instagram account had 10,449 followers. After 5 weeks of lockdown (20 April 2020), it had 33,836 followers, an increase of 223.8% (Figure 1).

## Discussion

Unlike the influencers studied above, the classes at the fitness club were given by fitness coaches who were presented as being qualified (Smith Maguire, 2001). These fitness teaching practices, which could be described as “digital coaching,” seemed, for those ensuring them, to fall within the framework of teleworking, a model of professional practice recommended by President Emmanuel Macron in his speech on 16 March.

However, the fact remains that this type of content, produced and posted by companies, such as FitnessSide for their Instagram followers (who were not limited to their clients), contributed to the construction of a digital space that multiplied incentives to work out at home. Thus, at a time when—health authorities were encouraging rest^10^, and when medical and healthcare personnel were facing an unprecedented health crisis, the social network Instagram saw the creation of a new dynamic in terms of fitness recommendations on the Internet that deserves to be examined^11^. This study thus intends to contribute to the existing literature on social media influencers and fitness. This article has shown that in France, the closure of sports facilities led to changes in the way people practice sport and social media have played an important role in this.

This paper identifies the different approaches in how individual fitness influencers and organizations used Instagram during the pandemic and contributed to producing knowledge to feed the debates around the impacts of COVID-19 on sport and active living and contribute to social media influence knowledge. By investigating the content published by influencers during the COVID-19 outbreak, this research shows that fitness influencers were mobilized during lockdown to promote physical exercise in the home. The legitimacy on which these individuals built their discourse originated in their physical appearance, which had been previously trained and staged on Instagram. Their bodies were seen as the guarantees of the advice given, far from a complete health control of the recommended exercises. At the same time, a chain of fitness clubs was using Instagram to offer its members continuity of service. The “live” sports sessions were provided by trained professionals. During this period, online physical training was more available than ever. The diversity of the content offered and delivered to Instagram users played an important role in providing opportunities for people to practice sport at home during lockdown.

Thus, the Instagram user is faced with digital sports content that serves the economic interests of influencers and a chain of fitness clubs. The sport “prosumer” can thus be considered to be linked with the conflation of play and labor known as “playbor” for the benefit of the digital capitalist class (Andrews and Ritzer, 2018, p. 368). In the same way to the institutional structure and operations of “prosumer sport 2.0” and eSports, influencers are characterized by abundant content production. The prosumer contributes to the creation of surplus value for the platform used. Within such conditions, the effectiveness of harnessing the prosumer's voluminous digital output suggests that influencers contribute to blurring the boundaries between labor and consumption (Jurgenson and Ritzer, 2009). Within this world of digital sport, the study of influencers' activities during lockdown shows contemporary sport culture can be considered both playground and factory where the digital user, the sport prosumer, is both entertained and exploited by sport prosumption (Scholz, 2013).

By reporting on strategies made visible on Instagram and most certainly initiated to limit financial losses, this paper aims to feed the debates relating to the “digitization” of fitness training during the COVID-19 lockdown and questions the consequences and the re-establishing of economic activities impacted by the governmental measures taken to deal with the pandemic.

## Data Availability Statement

The original contributions presented in the study are included in the article/supplementary material, further inquiries can be directed to the corresponding author/s.

## Author Contributions

The author confirms being the sole contributor of this work and has approved it for publication.

## Conflict of Interest

The author declares that the research was conducted in the absence of any commercial or financial relationships that could be construed as a potential conflict of interest.

## Footnotes

^1^This description should be viewed with a critical eye and is criticized by another part of this research project. Although Instagram is not a platform with a sales space, it nevertheless hosts a marketplace. Gradually learning this “self-marketing,” influencers on Instagram sell their image to brands.

^2^Each influencer was interviewed once. Semi-structured interviews lasted between 1 and 2 h and a half.

^3^All names in the data presented below are pseudonyms.

^4^The quotes from the French fitness influencers were translated from French by the first author.

^5^To guarantee the anonymity of the influencers, the names used in this paper are pseudonyms.

^6^Some respondents published up to one image per day and also used the “story” feature that allows content to be shared ephemerally.

^7^The number of followers on Instagram accounts is an indicator of visibility. However, it is very difficult to grasp the real scope of this audience. Furthermore, among these figures, some accounts may have been created from scratch with the sole purpose of inflating this figure, while others may have been created and no longer be consulted. In any case, even if this were to be ignored, the posts shared on Instagram are constrained by an algorithm that limits their distribution. In other words, it seems difficult to fully measure how many people in total received the messages disseminated by the individuals observed.

^8^As with the aforementioned influencers, in order to guarantee the anonymity of the company, “FitnessSide” is a fictitious name.

^9^A play on words with the French word “*confinement*,” meaning lockdown.

^10^On 20 March, some doctors said on the French TV news that it could be dangerous to practice exercise in a context of health crisis. They also adviced that sports should be avoided in order to avoid injuries and overburdening the hospitals. Robert Sebbag, a doctor attached to the department of infectious and tropical diseases at the Hôpital de la Pitié Salpêtrière, also affirmed that joggers accelerate the spread of the coronavirus. On March 22, 2020, the *Conseil d'Etat* (Council of State) asked the government to revise the travel exemption for physical activity. This was considered too broad.

^11^For example, Julie's Instagram account had 493,447 followers on 22 March 2020 (five days after the start of the lockdown). Six weeks later, there were nearly 100,000 additional followers (4 May 2020). The most significant increase was by 265,475 followers between 23 March and 27 April.

## References

[B1] Abdelnour S. Bernard S. (2018). Vers un capitalisme de plateforme ? Mobiliser le travail, contourner les régulations, in La nouvelle revue du travail, n°13. 10.4000/nrt.3734

[B2] Abdelnour S. Meda D. (2019). Les nouveaux travailleurs des applis, La vie des idées. Paris: PUF.

[B3] Abidin C. (2016). Visibility labour: engaging with influencers' fashion brands and #OOTD advertorial campaigns on instagram. Med. Int. Australia 161, 86–100. 10.1177/1329878X16665177

[B4] Abidin C. (2018). Internet Celebrity. Understanding Fame Online, Society Now. Bingley: Emerald Publishing.

[B5] Andrews D. Ritzer G. (2018). Sport and prosumption. J. Consum. Cult. 18, 356–373. 10.1177/1469540517747093

[B6] Beauvisage T. Beuscart J. S. Mellet K. (2018). Numérique et travail à-côté. Enquête exploratoire sur les travailleurs de l'économie collaborative. Sociol. Travail 60n°2, avril-juin. 10.4000/sdt.1984

[B7] Cardon D. Casillia A. (2015). Qu'est-ce que le Digital Labor? Bry-sur-Marne: INA, Coll. Etudes et controverses.

[B8] Cave A. Miller A. (2015). The Importance of Social Media in Sport. The Daily Telegraph. Available online at: http://www.telegraph.co.uk/investing/business-of-sport/social-media-in-sport/

[B9] Chaves Ferreira B. (2018). Les plateformes numériques révolutionnent-elles le travail? Une approche par le web scraping des plateformes etsy et la belle assiette. Réseaux 212, 85–119. 10.3917/res.212.0085

[B10] Détrez C. (2002). La construction sociale du corps. Seuil, Paris: Points Essais.

[B11] Hjorth L. Horst H. Bell G. Galloway A. (2017). Routledge Companion to Digital Ethnography. London: Routledge.

[B12] Jan A. (2018). Livrer à vélo… en attendant mieux. La Nouvelle Rev. Travail n°13. 10.4000/nrt.3803

[B13] Jourdain A. (2018). Faites de votre passion un métier. La Nouvelle Rev. Travail n°13. 10.4000/nrt.3870

[B14] Jurgenson N. Ritzer G. (2009). Efficiency, effectiveness, and web 2.0, in Culture of Efficiency: Technology in Everyday Life, ed Kleinman S. (New York, NY: Peter Lang), 51–67.

[B15] Luvaas B. (2012). DIY Style: Fashion, Music and Global Digital Cultures. Cambridge: Bloomsbury.

[B16] Mears A. (2011). Princing Beauty. The Making of a Fashion Model. London: University of California Press.

[B17] Miller D. (2011). Tales From Facebook. Cambridge: Polity Press.

[B18] Moreno Pestana J.-L. (2016). La classe du corps. Morale corporelle et troubles alimentaires. Limoges: Presses Universitaires de Limoge, Series: Sociologie & Sciences Sociales.

[B19] Ritzer G. Jurgenson N. (2010). Production, consumption, prosumption: the nature of capitalism in the age of the digital “prosumer”. J. Consum. Cult. 10, 3–36. 10.1177/1469540509354673

[B20] Scholz T. (2013). Digital Labor: The Internet as Playground and Factory. New York, NY: Routledge.

[B21] Smith Maguire J. (2001). Fit and flexible: the fitness industry, personal trainers and emotional service labor. Sociol. Sport J. 18, 379–402. 10.1123/ssj.18.4.379

[B22] Smith Maguire J. (2002). Body lessons: fitness publishing and the cultural production of the fitness consumer. Int. Rev. Sociol. Sport 37, 449–464. 10.1177/1012690202037004896

[B23] Smith Maguire J. (2008). The personal is professional: personal trainers as a case study of cultural intermediaries. Int. J. Cult. Stud. 11, 211–229. 10.1177/1367877908089265

[B24] Srnicek N. (2017). Platform Capitalism. Cambridge: Polity.

[B25] Wacquant L. (2004). Body & Soul: Notebooks of an Apprentice Boxer. Oxford: Oxford University Press.

[B26] Woodcock J. Graham M. (2018). Towards a fairer platform economy: introducing the fairwork foundation. Altern. Routes 29, 242–253.

[B27] Woodcock J. Graham M. (2020). The Gig Economy. A Critical Introduction. Cambridge: Polity.

